# Living with conduct problem youth: family functioning and parental perceptions of their child

**DOI:** 10.1007/s00787-017-1088-6

**Published:** 2017-12-04

**Authors:** Ruth Roberts, Eamon McCrory, Helene Joffe, Nicole De Lima, Essi Viding

**Affiliations:** 1 Division of Psychology and Language Sciences, University College London, 26 Bedford Way, London, WC1H 0AP UK; 2 School of Psychology, Cardiff University, Tower Building, 70 Park Place, Cardiff, CF10 3AT UK

**Keywords:** Conduct problems, Callous–unemotional traits, Family functioning, Qualitative methods, Adolescent males

## Abstract

Parenting children with conduct problems (CP) is challenging, yet very little is known about the impact of the child’s behaviour on family functioning or how parents of children with CP perceive their child. The aim of this research was to examine whether families with children with CP and high vs. low levels of callous–unemotional traits (HCU vs. LCU) experience differences in family functioning and parental perceptions. One hundred and one parents/caregivers of boys aged 11–16 [Typically developing (TD) *n* = 31; CP/HCU *n* = 35; CP/LCU *n* = 35] completed the McMaster Family Assessment Device, measuring multiple domains of family functioning. Parents/caregivers also completed a written statement describing their child, used for qualitative analysis. Families with CP/HCU children had poorer affective involvement than TD (*p* = 0.00; *d* = − 1.17) and CP/LCU (*p* = 0.03; *d* = − 0.62) families. Families with CP/HCU children showed significantly poorer general family functioning (*p* = 0.04; *d* = − 0.63) and more poorly defined family roles (*p* = 0.005; *d* = − 0.82) than families with TD children. Qualitative analyses indicated that parents/caregivers of CP/HCU children characterised them as having a dichotomous personality and being superficially charming. CP/LCU children were characterised as cheeky and endearing, with parents reporting good rapport. Families with CP/HCU children presented with specific difficulties in affective involvement and parents described challenges which were in line with the child’s specific presentation of lack of empathy and shallow affect. These findings may be used to help clinicians identify targets for family interventions.

**Electronic supplementary material:**

The online version of this article (10.1007/s00787-017-1088-6) contains supplementary material, which is available to authorized users.

## Introduction

Conduct problems (CP) refer to a range of challenging behaviours including bullying, physical cruelty towards people and animals, and serious disregard for rules and norms [36]. Children with CP have an increased likelihood of adverse adult outcomes and represent a substantial cost to society in terms of health service provision, specialist schooling, and social services [26]. The combination of increased service use, poor adult outcomes, and challenging daily behaviour combine to create a significant societal burden and emotional toll on those around them.

The fifth edition of the Diagnostic and Statistical Manual of Mental Disorders DSM-5; [2] has added a ‘Limited Prosocial Emotions’ specifier to identify a subgroup of CP children who display low levels of empathy and remorse and persistent aggressive behaviour, consistent with the presence of high levels of callous–unemotional (CU) traits (CP/HCU) [38]. CP/HCU children show deficits in processing negative emotional stimuli and low reactivity to fear and distress signals in others [4]. Children with CP and low levels of CU traits (CP/LCU), on the other hand, do not display deficits in empathy and guilt but are impulsive and display more reactive aggression to perceived threats which can leave them feeling anxious about the outcomes of their behaviour [4].

A growing body of research indicates that children with CP evoke very different parenting reactions than their non-CP peers and adoption studies have unequivocally demonstrated that those children at higher biological risk for antisocial behaviour are more likely to evoke suboptimal parenting responses than their adoptive peers without biological risk for antisocial behaviour [10, 25, 30]. This strongly suggests that children with or at risk of developing CP present their families with unique challenges. Much of the research into CP and CU, including that which has examined the bidirectional nature of parent–child relationships, has focussed on parenting variables [19, 21, 28]. Harsh parenting, as well as negative and inconsistent discipline have been associated with higher CU traits in children, while warm and positive parenting has been shown to help decrease CU traits in children [39]. While a large body of parenting research exists, less work has concentrated on exploring family functioning, which examines various aspects of family life rather than just the parent–child relationship. Children with CP (including those with HCU) are more likely to come from homes characterised by chaos, discord, and less than optimal parenting strategies [11, 32]. Children with CP engage in aggressive and violent behaviour, which causes emotional stress and discord in the family [12, 16, 25]. They often resist being told what to do and have difficulty following rules [32], which can cause frustration for parents and may contribute to family systems breaking down [16, 32]. Given that both parenting and home environment contribute to the development of CP and CU, exploring family functioning has potential to provide additional insight into how the family functions as a whole.

George et al. [16] conducted one of the first studies examining the family environment of children with CP using an instrument called the Family Environment Scale that measures family structure and interactions, and community involvement. They found CP in children was associated with low family cohesion and high levels of family conflict [16]. ‘Unhealthy’ family functioning, as measured by the ‘general functioning’ subscale score of the McMaster Family Assessment Device (FAD; [6]) has also been found to be associated with CP in large community samples [1, 34]. Despite the emerging evidence base regarding problematic family functioning in families with children with CP, no extant studies have systematically examined family functioning in families who have children with CP/HCU and CP/LCU as compared with typically developing (TD) controls. Previous studies have only focused on specific domains of family functioning, rather than assessing a more comprehensive set of indicators. Additionally, children with CP often present with comorbidities that have not previously been considered in studies of CP and family functioning.

There are reasons to suspect that HCU and LCU groups, despite both displaying CP, may not have identical profiles of family functioning. Decades of research indicate that children with CP/HCU are generally insensitive to punishment and overestimate the positive outcomes of their antisocial actions [27, 29]. They may also value the instrumental benefits of their action above any possible consequence [29], making them relatively unlikely to consider possible sanctions and particularly challenging to parents. CP/HCU children also show a reduced desire to please others [13] so parents and family members may feel less connection with the child if they are not able to share positive outcomes together. CP/LCU children, on the other hand, are able to feel empathy and guilt [4, 37]. Their aggression is often impulsive and triggered by perceived threats or frustration [4, 37]. CP/LCU children do not always accurately predict the consequences of their choices and behaviour [4, 37], which may make it more difficult for parents to control their behaviour.

We collected data on multiple domains of family functioning using the FAD (e.g. assessing family functioning beyond ‘unhealthy’ general functioning) while controlling for various comorbidities of CP. This facilitated a more nuanced picture of family functioning that may be related to having a CP child in general vs. a child with CP/HCU traits more specifically. We also collected short, written parental descriptions of the child to conduct exploratory qualitative analyses on parents/caregivers’ perceptions of their child. Qualitative research on parenting children with CP is surprisingly rare, thus lending little insight into the nature of the struggles that parents of children with CP face on a daily basis. In a recent study by Lewis et al. [22] examining how parents ‘made sense’ of their child with CP, parents describe difficulties in dealing with their child’s emotional behaviour, as well as the impact that the child’s behaviour had on their own emotions. Webster-Stratton et al. [40] qualitatively explored the effects that the CP child had on the family unit and found challenges with siblings and family and marital discord, as well as challenges faced by the family in the community. While the experiences of a small group of parents may not be generalisable to all parents of children with CP, they offer insight into how parents of children with CP may think about and cope with their child. No published studies have focused on how qualitative descriptions of a child with CP may differ as a function of the child displaying high vs. low levels of CU traits. We asked parents to *spontaneously* describe their child which enabled us to further characterise family functioning in families of children with CP/HCU and CP/LCU.

We hypothesised that the CP/HCU group would have poorer functioning than both TD and CP/LCU groups on the affective involvement and affective responsiveness scales of the FAD in light of the child’s pervasive lack of empathy and diminished tendency to seek affiliation and approval. We hypothesised that families with children with CP would have poorer general functioning as compared to their TD peers due to the disruptive behaviour that CP children exhibit on a daily basis and the impact that managing this behaviour would have on family ecology. Due to their tendency to react with impulsive aggression to perceived threats, we predicted that CP/LCU would have poorer functioning on the behaviour control scale than TD groups but possibly look similar to CP/HCU as both groups share the ability to be aggressive and violent when frustrated. The qualitative analysis of parent’s perception of their CP/HCU and CP/LCU children was exploratory in nature.

## Methods

### Participants

One hundred and one boys aged 11–16 years of age and their parent/caregiver were recruited from the community in the greater London area, via newspaper advertisements and both mainstream schools and schools who catered for pupils with behavioural difficulties. Participant characteristics are displayed in Table 1. The full research protocol was approved by the University College London Research Ethics Committee (Project ID number: 0622/001). Parents/caregivers and the children were provided detailed information sheets outlining the aims of the study and what participation entailed, and given an opportunity to ask questions and seek clarification on any aspect of the study about which they were unsure. Children were also provided with detailed information sheets about the research, written in age-appropriate language. Parents/caregivers provided written informed consent and assent to participate was obtained from all children. Researchers were trained by an experienced clinician on how to sensitively interact with participants with conduct problems and their families. The clinician was available to advise on any concerns over the duration of the project. Exclusion criteria for child participants included a diagnosis of autism or Asperger’s syndrome, any reported neurological abnormality, and cognitive ability of < 70 on a standardised cognitive assessment. No exclusion criteria were applied for parents.

**Table 1:** Demographic data

Characteristics and questionnaires	TD controls (*n* = 31)			CP/LCU (*n* = 35)			CP/HCU (*n* = 35)				*p* value^a^	Post hoc*
	Mean	SD	Range	Mean	SD	Range	Mean		SD	Range		
Child age (years)^b^	13.96	1.85	5.85	14.56	1.54	5.60	14.66		1.41	5.54	0.17	
Child IQ (full score, two-subtest WASI)^c^	100.68	12.72	51	101.06	14.16	54	95.35		12.34	58	0.14	
Child ethnicity^b,f^	4:17:10			5:21:9			6:20:9				0.96	
ICU^d^	25.39	7.36	33.00	33.61	7.39	32.00	51.00		6.57	26.00	0.00	1 < 2 < 3
CASI conduct disorder^d^	0.58	0.76	2.00	6.00	3.58	19.00	11.02		4.97	21.00	0.00	1 < 2 < 3
CASI attention deficit hyperactivity disorder^e^	11.31	6.73	31.00	23.07	11.86	41.00	25.54		11.84	49.00	0.00	1 < 2/3
CASI generalised anxiety disorder^e^	3.63	2.02	7.00	8.60	5.00	18.00	9.05		4.08	18.00	0.00	1 < 2/3
CASI major depressive episode^e^	2.99	1.50	5.78	6.20	3.95	13.00	6.84		4.31	15.00	0.00	1 < 2/3
Alcohol use and disorders^c^	0.60	1.53	6.75	2.81	5.50	22.00	2.30		3.78	18.00	0.08	
Drug use and disorders^c^	0.13	0.72	4.00	2.44	3.56	11.00	2.00		4.32	20.00	0.02	1 < 2
SES^b^	2.87	1.12	4.00	2.67	1.14	4.00	3.15		0.83	3.00	0.15	
Parent/caregiver informant^e,g^	23:3:0			29:5:1			32:0:3				1.00	
Family structure^b,h^	15:16			19:16			15:20				0.63	
Child birth order^e,i^	15:9:4:3			14:11:4:4			14:14:5:1				0.83	
Number of people living in household^e,j^	4:10:9:6:2			0:9:16:6:3			7:8:8:6:6				0.11	
Parent self-report psychopathy^b^	11.36	9.66	39.74	11.57	9.76	43.00	14.15		9.87	39.00	0.43	

*TD* typically developing, *CP/LCU* conduct problems and low levels of callous-unemotional traits, *CP/HCU* conduct problems and high levels of callous-unemotional traits, *SD* standard deviation, *WASI* Wechsler Abbreviated Scale of Intelligence, *ICU* inventory of callous-unemotional traits, *CASI* Child and Adolescent Symptom Inventory, *SES* socio-economic status

* *p* < 0.05, Bonferroni corrected

^a^All *p* values obtained using analysis of variance, except child ethnicity and family structure (Chi-square) and parent/caregiver informant, child birth order, number of people living in the household (Fisher’s exact test)

^b^Measures obtained at screening phase, parent report

^c^Measures obtained at testing session, child report

^d^Measures obtained at screening phase, parent and teacher report

^e^Measures obtained at testing session, parent report

^f^Counts for each ethnicity (Black:White:Mixed/Asian)

^g^Counts for each rater category [Mother:Father:Other (Foster/Adoptive/Grandparent)]

^h^Counts for two-parent/carer household:single-parent/carer household

^i^Counts for 1st:2nd:3rd:4th born

^j^Counts for family size of 2:3:4:5:6 + family members

### Measures

#### Screening

Screening questionnaires assessing CP, CU traits, and psychopathology were completed by parents/caregivers and teachers to ascertain CP/HCU, CP/LCU, and TD comparison groups. Screening measures were scored by taking the highest ratings from either the parent or teacher questionnaire for each item [33]. For eleven children with CP, only parent ratings were available at screening. All parents/caregivers provided further information pertaining to psychiatric diagnoses and demographics after screening, once they had been successfully recruited for the study.

CP was assessed using the *Child and Adolescent Symptom Inventory* (CASI-4R; [15]) Conduct Disorder scale (CASI-CD). CASI-CD cut-off scores for inclusion in the CP group were as follows: parent report = 4 + (ages 10–12) and 3 + (ages 13–16) or teacher report = 3 + (ages 10–12), 4 + (ages 13–14), and 6 + (ages 15–16). These scores are associated with a clinical diagnosis of CD [14]. Seventy children meeting the screening criteria for CP were recruited for this study. CU traits were assessed using the *Inventory of Callous*-*Unemotional Traits* (ICU), a widely used instrument for quantifying CU traits [9]. A median split of the ICU scores for the children meeting CP criteria was used to determine assignment to CP/HCU and CP/LCU groups. Thirty-five children met CP/LCU criteria with ICU scores less than or equal to 42 and thirty-five children met criteria for CP/HCU with ICU scores greater than 42. The *Strengths and Difficulties Questionnaire* (SDQ; [17]) was used to screen for psychopathology in the control participants. Thirty-one children met screening criteria for typically developing controls, scoring less than 42 on the ICU, less than or equal to 2 on the CASI-CD, and within the normal range on the SDQ subscales. For all groups, exclusion criteria included diagnosis of autism or Asperger’s syndrome, diagnosis of a neurological or psychotic disorder, and use of prescription medication for behavioural difficulties.

Parents/caregivers were not subject to any selection criteria but provided information about child birth order, number of parents/caregivers in the household (biological, stepparents, foster and adoptive parents, grandparents), total number of people living in the household, and completed the Self-Report Psychopathy-Short Form (SRP-SF, [18]) to assess parent/carer psychopathy (Table 1). Parents/caregivers also provided information about parental education and employment to ascertain family socio-economic status (SES).

#### The McMaster Family Assessment Device (FAD; [8])

The FAD is a 60-item self-report measure of family characteristics and functioning. The FAD has been found to be a valid and reliable measure of healthy and unhealthy family functioning [23]. The FAD is comprised of seven subscales, which assess problem-solving, communication, roles, affective responsiveness, affective involvement, behaviour control, and general functioning ([8, 24]; see Online Resource 1 for details of the measure). Participants (parents/caregivers in this case) rate how well each statement describes their own family. Higher scores indicate worse levels of functioning.

#### Qualitative component: parental description of their child

Parents/caregivers were asked to describe the child with open-ended, written responses to the following question: *Please describe your child* (*no specific type of description is required*, *you should just write whatever comes into your head*). They were given as much time as they needed to complete the question and were not restricted in the length of their response.

#### Additional measures

Child participants completed the *Wechsler Abbreviated Scale of Intelligence* (WASI; [41]) two-subtest version, to assess cognitive ability. The *Alcohol Use Disorder Identification Test* (AUDIT; [35]) and the *Drug Use Disorder Identification Test* (DUDIT; [3]) (Table 1) were completed by the child participants to assess substance use. Parents/caregivers completed the CASI-4R scales for conduct disorder (CD), attention deficit hyperactivity disorder (ADHD), generalised anxiety disorder (GAD), and major depressive episode (MDE) to assess for commonly occurring comorbidities with CP (Table 1).

### Procedure

Parents completed the FAD to assess family functioning and provided a written qualitative description of their child. For the qualitative description of the child, researchers explained to the parent/caregivers that they should simply write what first came to mind when thinking about their child; there were no expectations by the researchers regarding what the parents should say. In two cases, the parent was unable to write (owing to literacy problems and disability) and their verbal responses were recorded by a trained researcher. Written qualitative data collection has distinct advantages which were of benefit to our participants: (1) this method provided parents an extra layer of anonymity so they could be free to give honest answers about their child and, (2) this method gave parents time to reflect upon their answers and not feel rushed in their responses [7].

## Statistics

### Demographics

To explore demographic characteristics of the groups, analysis of variance (ANOVA) was computed to compare differences between the means for child age, child IQ, child alcohol and drug use, child CU traits, CASI conduct disorder, ADHD, GAD and MDE subscales, family SES, and parental self-reported psychopathy. Chi-square was computed to compare child ethnicity and family structure. Fisher’s exact test was computed to examine whether groups differed on parent/caregiver informant, childbirth order, and total number of people living in the household. Bonferroni corrections were computed for multiple comparisons. As CP often co-occur with substance use and other psychopathologies, analysis of covariance (ANCOVA) was computed to control for common comorbid disorders (ADHD, GAD, MDE, alcohol and drug use).

### McMaster Family Assessment Device (FAD)

A one-way ANOVA was conducted to determine if the groups differed on the FAD subscales. For those subscales showing overall significant group differences, Tukey’s post hoc analyses were conducted to examine the differences between groups. Effect sizes were computed to quantify the difference between the groups.

### Qualitative analysis of parental description of the child

Prior to conducting the content analysis, data entry was checked for accuracy and completeness of participant statements. Any identifiable data (names) were removed. Content analysis [5] was used to explore the raw data and help to identify the most prevalent parental perceptions about their child. The prevalent perceptions in the content analysis were used to inform further thematic analysis [5] of the statements. Researchers read through the statements multiple times, and notes were made about the patterns and points of interest in the parental description of their child. These patterns were assembled to form a coding frame (see Online Resource 2 for qualitative theme overview). Codes were clustered into overall themes by exploring the relationship between the codes and the code’s relevance to the child’s group assignment. To assess the reliability of the coding frame a second rater double coded all of the interview transcripts and Cohen’s Kappa was computed to check agreement between raters. Discrepancies were discussed and resolved between raters.

## Results

No differences were found between groups on child age, child IQ, child alcohol use, SES, child birth order, total number of people living in the household, and parental psychopathy. There was no difference between groups on child ethnicity, family structure and parent informant. The TD group was lower on clinical indicators than both CP groups. The CP/HCU group had higher scores on conduct problems and callous-unemotional traits than the CP/LCU group as per group assignment. CP/HCU and CP/LCU groups did not differ significantly on the CASI ADHD, GAD, MDE, scales or AUDIT scores but did differ on DUDIT scores (Table 1).

### McMaster Family Assessment Device

#### Affective involvement

There was an overall group difference on the affective involvement subscale, *F* (2, 91) = 11.70, *p* = 0.000. Post hoc analyses revealed significant differences between TD and CP/HCU with a large effect size (*p* = 0.00; *d* = − 1.17) and CP/LCU and CP/HCU with a medium effect size (*p* = 0.03; *d* = − 0.62). TD and CP/LCU did not differ significantly but had a medium effect size (*p* = 0.057; *d* = − 0.69). The effect of group on affective involvement remained statistically significant after adjusting for child ADHD, GAD, MDE, and substance use (see Online Resource 3 for covariate analysis).

#### General functioning

There was an overall group difference on the general functioning subscale, *F* (2, 93) = 3.32, *p* = 0.04. Post hoc analyses revealed significant differences between TD and CP/HCU with a medium effect size (*p* = 0.04; *d* = − 0.63). No other group differences were statistically significant. After adjusting for child ADHD, GAD, MDE and drug use, the effect of group on general functioning was no longer significant (Online Resource 3).

#### Roles

There was an overall group difference on the roles subscale, *F* (2, 92) = 5.40, *p* = 0.006. Post hoc analyses revealed significant differences between TD and CP/HCU with a large effect size (*p* = 0.005; *d* = − 0.82). TD and CP/LCU did not differ significantly but had a medium effect size (*p* = 0.055; *d* = − 0.63). There were no significant differences between the two CP groups. After adjusting for child ADHD and GAD, the effect of group on roles was no longer significant (Online Resource 3).

As two items in the roles subscale potentially overlap with CP behaviours (“When you ask someone to do something, you have to check that they did it” and “If people are asked to do something, they need reminding”) analyses were also conducted without those two items. The overall group difference remained significant, *F* (2, 93) = 4.31, *p* = 0.016, with post hoc analysis revealing significant differences between TD and CP/HCU (*p* = 0.018, *d* = 0.70). As before, there was no difference between the two CP groups, and the effect of group on roles was no longer significant after adjusting for ADHD and GAD.

There were no statistically significant differences between the groups on the problem solving, communication, affective responsiveness, and behavioural control scales on the FAD (*p* values = 0.12–0.99; Online Resource 3).

### Qualitative analysis of parent descriptions of CP/HCU and CP/LCU children

Cohen’s Kappa revealed a ‘substantial’ agreement between raters, *κ* = 0.65, *p* = 0.000 [20]. As the focus of the qualitative analysis was to understand parental perceptions of CP/HCU and CP/LCU children, TD qualitative data is not presented in this paper. Themes within the parent/caregiver descriptions of the child are presented on the basis of their relevance to the child’s group assignment and connectedness to the FAD. Qualitative themes included the dichotomous nature of the CP/HCU child, the perception of the CP/LCU child as a cheeky and loveable character, and a greater sense of rapport between parents and CP/LCU children (Online Resource 2).

### Dichotomous child

#### Theme 1: changeable moods

Parents/caregivers of CP/HCU children frequently described their child as being unpredictable and changeable. The dichotomous nature of their child’s personality was of concern to many parents/caregivers of CP/HCU children, who described their child as ‘*loving*’ and ‘*bubbly*’ but could turn ‘*opposite*’ or ‘*dark*’ when stressed or provoked. One parent described being in a state of vigilance over their child’s moods: “*…sometimes it’s like living with a volcano waiting for it to explode…*”. Parents/caregivers of CP/LCU children also described their child as capable of variable moods but did not seem to have the same challenges with extreme and unpredictable moods as CP/HCU parents.

#### Theme 2: instrumental charm

In keeping with the child’s changeable nature, CP/HCU parents/caregivers also reported that their child could switch on the charm to gain something from them: “*…can be very charming when he needs something from you*”. A charming persona was employed when it suited the child and could be turned on and off at will. Parents/caregivers of CP/LCU children on the other hand did not describe any premeditation in their child’s expressions of kindness. For example, one CP/LCU parent describes her child as “*…capable of spontaneous kindness and sympathy*”.

### Cheeky child

#### Theme 1: normalising of behaviour

While CP/HCU parents/caregivers saw their children’s behaviour as fundamentally problematic, many CP/LCU parents/caregivers offered explanations that appeared to minimise the seriousness of their child’s behaviour. For example, many described their child in a playful tone, such as using the word ‘*cheeky*’ or the term “*cheeky chappie*”. This gave the sense that CP/LCU parents/caregivers normalised their child’s behaviour seeing it as endearing, and typical behaviour for a teenage boy. CP/LCU parents/caregivers were also more likely to identify ‘reasonable’ triggers for the child’s behaviour or attribute it to characteristics that might be unusual but not problematic; for example, one CP/LCU parent described their child as having “*an interesting and quirky personality*”.

#### Theme 2: warmth and affection

CP/LCU parents/caregivers commonly described their child as a loveable and loving character. The words “*loving*”, “*caring*”, and “*lovely*” were frequently used by CP/LCU parents to describe their child. CP/LCU parents/caregivers often described their child as being “*funny*” or having a “*good sense of humour*”. This is not to say that CP/LCU parents/caregivers did not discuss some serious challenges in their child’s behaviour or that CP/HCU parents/caregivers were completely devoid of affection towards their children, but there was an overall sense of a closer, more affectionate relationship between parent/caregiver and child in the CP/LCU group as compared to CP/HCU group.

### Rapport with child

Both CP/LCU and CP/HCU parents/caregivers gave rich descriptions of their children, however, CP/LCU parents/caregivers tended to characterise their child’s personality and mental state, thinking about what their child might be going through (e.g. “*He is very resilient given the changes in his life that he has experienced*”), whereas CP/HCU parents/caregivers were more inclined to focus on their child’s behaviours. CP/LCU parents/caregivers described their child as “*intelligent*” or “*clever*” more frequently than CP/HCU parents/caregivers despite there being no significant difference in the average IQ scores of the two groups of children.

## Discussion

Families of children with CP/HCU functioned less well than both TD and CP/LCU families with qualitative results providing some insight into what may be contributing to the differences in family functioning. Families of children with CP/HCU had poorer functioning on the affective involvement scale than both TD and CP/LCU families and presented with poorer functioning on both the general functioning and roles scales than families with TD children. The exploratory qualitative analyses were in line with the findings obtained from the quantitative assessment of the FAD and provided insight into how parents of children with CP perceive their child.

CP/HCU families differed significantly from the TD as well as CP/LCU families on the affective involvement subscale of FAD, although the groups did not differ on affective responsiveness as predicted. The affective involvement subscale best quantifies aspects of family functioning that correspond with the CP/HCU profile, including using others for personal gain and looking out for number one with items such as: “We only show interest in each other when we can gain something out of it personally”, and “We are too self-centered”. This finding is supported by qualitative reports from parents/caregivers of CP/HCU children who described their child as being able to switch on a charming persona if they wanted to gain something from them. The difference between CP/LCU and TD families approached significance and was of medium effect size suggesting that difficulties with affective involvement may also be a problem for families with CP/LCU children. However, qualitative analysis found that CP/LCU parents/caregivers had a warm relationship and good rapport with their child (which was not as clearly demonstrated by CP/HCU parents), which may ameliorate challenges in this domain. Interestingly, the effect of group remained significant for affective involvement even after controlling for various CP comorbidities. The robustness of this finding, alongside the qualitative descriptions, indicates that a child with CP/HCU (primarily preoccupied with his own needs) can have a substantial negative impact on the way in which families are able to function collaboratively.

Families with CP/HCU children also had poorer general functioning than TD families. Parents/caregivers of CP/HCU children qualitatively described their child as being unpredictable and changeable which left families feeling uneasy. While affect is known to be shallow in children presenting with CP/HCU, their parents/caregivers nonetheless describe strong emotional reactions that occur when the child is stressed about not getting his way. Although CP/LCU children also present with challenging and difficult behaviour, CP/LCU parents/caregivers seemed more able than CP/HCU parents/caregivers to normalise some of this behaviour and see their child as a loveable and endearing character. They also seemed to have a greater understanding of their child’s challenges. This rapport and understanding of their child could be why CP/LCU parents/caregivers did not differ significantly from TD parents on general functioning or the behaviour control subscale of the FAD as predicted. Research has also shown that as parents increase discussion about child behaviour problems with their CP/LCU child, the behaviour problems get worse (reactive aggression) so they may speak less about the child’s naughty behaviour to illicit less negative reactions in the child [31]. The effect of group on general functioning did not remain significant, however, after controlling for ADHD, GAD, MDE, and drug use. Previous studies have found worse general functioning in CP families than TD families but did not systematically control for comorbidities of CP [1, 34]. Further research into the effect of CP on family functioning is warranted.

CP/HCU families functioned significantly less well than TD families in terms of their ‘roles’ which examines how families fulfil functions and responsibilities with items such as: “When you ask someone to do something, you have to check that they did it”. The difference between CP/LCU and TD families approached significance and was of medium effect size. It, therefore, appears that the CP/HCU group is the most impaired on this dimension of family functioning, but that the CP/LCU group may not be entirely typical either. The observed difference could be due to children with CP not caring about pleasing others and requiring more cajoling regarding completing household tasks. CP children only want to do things on their own terms so they may not be compliant with requests if it does not suit them. The overall group difference remained significant even after removing two items from the roles scale that potentially overlapped with CP behaviours. This suggests that difficulties with roles go beyond those social interactions that directly reflect CP presentation. Future longitudinal research could formally investigate this finding further, for example examining whether time spent managing CP behaviours infringes on more normative activities and social interactions. As the groups did not differ significantly on number of parents/caregivers and SES, the worse functioning in the roles domain was thus not likely due to lack of parental or family resources but more likely owing to child driven factors. The effect of group on roles was no longer significant after controlling for ADHD and GAD but this is not surprising given that parents/caregivers may not feel confident in assigning tasks to children who are unfocussed or anxious.

There are a number of limitations that should be noted. First, the sample selection was based around child rather than parent/caregiver characteristics, with parents/caregivers reporting similar levels of resources and self-reported psychopathy. While this aspect of the study limits the generalisability of the findings it offers an opportunity to look at the impact of child behaviour on family functioning in families with similar resources and parent/caregiver characteristics. Future research should seek to examine the family environment and parenting domains in parents who have higher levels of antisocial behaviour and higher material needs to gain a more complete picture of family functioning in families with children who have CP. Second, the study focused on males only, given the preponderance of CP in boys and the desire to maximise the statistical power. It would be of interest to investigate family functioning in families of girls with CP in the future. Finally, this research is not able to determine the direction of the relationship of family functioning and CP/CU over development. Future research would benefit from having a child report of family functioning to elucidate specific parent and child influences on family functioning, particularly within a longitudinal framework.

The strength of the current study is the inclusion of both qualitative and quantitative forms of assessment. Given the rich data provided by parents in response to the request to describe their child, future research might consider a full qualitative interview with multiple family members living with children who have CP. The current data can be helpful for clinicians in their work with children presenting with CP and their families. For those cases where a child presents with CP/HCU, the clinician can be mindful that parents may see their child’s behaviour as instrumental and unpredictable. In addition, they may wish to attend to improving affective involvement of the whole family, promoting an interest in each other’s interests and priorities. For those cases where a child presents with CP/LCU, the clinician can be mindful that parents may tend to normalise their child’s behaviours and reframe possible difficulties as endearing rather than problematic. In this way, these findings can help clinicians build a priori formulations of family functioning in children presenting with CP. This may help shape how an intervention is framed and introduced for this group, particularly in light of the frequent difficulties in engaging parents and children in treatment.

This research provides additional context to the existing research on parenting children with CP and demonstrates that families with CP/HCU children experience challenges with affective involvement even when controlling for CP comorbidities and when family resources and parent characteristics are similar to those of families of TD children. Families with CP/LCU children experience fewer challenges with family functioning, which could be due to parents/caregivers having a good rapport with their child.

## Electronic supplementary material

Below is the link to the electronic supplementary material.

Supplementary material 1 (PDF 6 kb)

Supplementary material 2 (PDF 6 kb)

Supplementary material 3 (PDF 11 kb)
