# Supplementary material for: Living with conduct problem youth: family functioning and parental perceptions of their child
Source: Eur Child Adolesc Psychiatry. 2017 Dec 4;27(5):595–604. doi: 10.1007/s00787-017-1088-6 (PMC5945745; doi:10.1007/s00787-017-1088-6)
Supplement: Supplementary file 1 — Supplementary material 1 (PDF 6 kb) [file 787_2017_1088_MOESM1_ESM.pdf]

Living with conduct problem youth: Family functioning and parental perceptions of their child

Ruth Roberts[1*], Eamon McCrory[1], Helene Joffe[1], Nicole De Lima[2] & Essi Viding[1]

[1]Division of Psychology and Language Sciences, University College London, 26 Bedford Way, London WC1H 0AP, UK

[2]School of Psychology, Cardiff University, Tower Building, 70 Park Place, Cardiff, CF10 3AT, UK

*Corresponding author:

Ruth Roberts

Email: r.roberts@ucl.ac.uk

Online resource 1. *Description of the McMaster Family Assessment Device scales*

Subscale	Domain
Problem solving	The family's ability to resolve problems
Communication	The way in which the family exchanges information
Roles	How individuals fulfil family functions and responsibilities
Affective responsiveness	The family's ability to respond to others and events with a range of appropriate actions and emotions
Affective involvement	The degree of interest in and value shown toward other family member's activities and interests
Behaviour control	The pattern that the family employs for behaviour management
General functioning	A composite measure of overall family functioning

Epstein NB, Baldwin LM, Bishop DS (1983) The McMaster Family Assessment Device. J Marital Fam Ther 9:171-180

Miller I, Ryan C, Keitner G, Bishop D, Epstein N (2000) The McMaster approach to families: theory, assessment, treatment and research. J Fam Ther 22:168-189
