# Supplementary material for: Living with conduct problem youth: family functioning and parental perceptions of their child
Source: Eur Child Adolesc Psychiatry. 2017 Dec 4;27(5):595–604. doi: 10.1007/s00787-017-1088-6 (PMC5945745; doi:10.1007/s00787-017-1088-6)
Supplement: Supplementary file 2 — Supplementary material 2 (PDF 6 kb) [file 787_2017_1088_MOESM2_ESM.pdf]

Living with conduct problem youth: Family functioning and parental perceptions of their child

Ruth Roberts[1*], Eamon McCrory[1], Helene Joffe[1], Nicole De Lima[2] & Essi Viding[1]

[1]Division of Psychology and Language Sciences, University College London, 26 Bedford Way, London WC1H 0AP, UK

[2]School of Psychology, Cardiff University, Tower Building, 70 Park Place, Cardiff, CF10 3AT, UK

*Corresponding author:

Ruth Roberts

Email: r.roberts@ucl.ac.uk

Online resource 2. *Qualitative themes for parental descriptions of their child*

Group	Theme	Codes	Example
HCU	Dichotomous Child	Changeable moods	"Overall we describe him as a complete contradiction, enchanting one minute and unbearable the next."
		Instrumental charm	"He can switch back to being extremely nice and charming when it suits him and most of the time this is due to him wanting something."
LCU	Cheeky Child	Normalising behaviour	"He can be aggressive towards me only at times and has had some counselling for this."
		Warmth & affection	"___ is very kind, caring and not afraid to show it."
	Rapport with Child	Characterisation of child	"_____ is interested and reflective of the world around him"
