# Supplementary material for: Living with conduct problem youth: family functioning and parental perceptions of their child
Source: Eur Child Adolesc Psychiatry. 2017 Dec 4;27(5):595–604. doi: 10.1007/s00787-017-1088-6 (PMC5945745; doi:10.1007/s00787-017-1088-6)
Supplement: Supplementary file 3 — Supplementary material 3 (PDF 11 kb) [file 787_2017_1088_MOESM3_ESM.pdf]

Living with conduct problem youth: Family functioning and parental perceptions of their child

Ruth Roberts[1*], Eamon McCrory[1], Helene Joffe[1], Nicole De Lima[2] & Essi Viding[1]

[1]Division of Psychology and Language Sciences, University College London, 26 Bedford Way, London WC1H 0AP, UK

[2]School of Psychology, Cardiff University, Tower Building, 70 Park Place, Cardiff, CF10 3AT, UK

*Corresponding author:

Ruth Roberts

Email: r.roberts@ucl.ac.uk

Online resource 3. *Covariate analysis for conditions comorbid with conduct problems*

						ANCOVA										
	ANOVA		Tukey post hoc			ADHD		GAD		MDE		AUDIT		DUDIT		
FAD subscales	F	*p*	Group	*p*	*d*	F	*p*	F	*p*	F	*p*	F	*p*	F	*p*	*post hoc*
Affective involvement	11.704	**0.000**	TD – HCU	**0.000**	-1.170	5.078	**0.008 (TD-HCU 0.008)**	5.263	**0.007 (TD-HCU 0.012)**	7.565	**0.001 (TD-HCU 0.001)**	10.902	**0.000 (TD-HCU 0.000)**	11.043	**0.000 (TD-HCU 0.000)**	**1\2 >3**
			TD – LCU	0.057	-0.685											
			HCU – LCU	**0.028**	-0.620				**(HCU-LCU 0.042)**		**(HCU-LCU 0.038)**		**(HCU -LCU 0.030)**		**(HCU -LCU 0.027)**	
General functioning	3.319	**0.041**	TD - HCU	**0.040**	-0.628	0.857	0.428	0.481	0.619	2.022	0.138	3.248	**0.043 (TD-HCU 0.049)**	2.943	0.058	
			TD – LCU	0.139												
			HCU – LCU	0.839												
Roles	5.399	**0.006**	TD – HCU	**0.005**	-0.818	3.098	0.050	2.695	0.073	3.898	**0.024 (TD-HCU 0.023)**	4.847	**0.010 (TD-HCU 0.009)**	4.550	**0.013 (TD-HCU 0.011)**	
			TD – LCU	0.055	-0.633											
			HCU – LCU	0.650												
Problem solving	2.193	0.117				1.416	0.248	1.095	0.339	2.774	0.070	2.533	0.085	1.931	0.151	
Communication	0.048	0.953				0.254	0.776	0.224	0.800	0.088	0.916	0.099	0.906	0.017	0.983	
Affective responsiveness	1.104	0.336				1.178	0.313	0.766	0.468	0.791	0.457	1.668	0.194	1.855	0.162	
Behaviour control	0.008	0.992				0.015	0.985	0.073	0.930	0.009	0.991	0.022	0.978	0.023	0.977	

TD= typically developing; HCU = high levels of callous-unemotional traits (and conduct problems); LCU = low levels of callous-unemotional traits (and conduct problems); FAD = McMaster Family Assessment Device; ADHD = Attention deficit hyperactivity disorder; GAD = Generalised anxiety disorder; MDE = Major depressive episode; AUDIT = Alcohol Use Disorders Identification Test scores; DUDIT = Drug Use Disorders Identification Test scores.

There is a statistically significant effect of group on affective involvement even after controlling for the following covariates: ADHD, GAD, MDE, AUDIT, DUDIT. Post hoc analysis indicates a significant difference between the TD and HCU groups and the HCU and LCU groups except when controlling for ADHD where there is no significant difference between HCU and LCU groups.

There is no statistically significant effect of group on general functioning when controlling for ADHD, GAD, MDE or DUDIT. There is no statistically significant effect of group on roles when controlling for ADHD or GAD.

There is no statistically significant effect of group on problem solving, communication, affective responsiveness, and behaviour control after controlling for any of the following covariates: ADHD, GAD, MDE, AUDIT, DUDIT.
